# Inhalant use and psychosis: EXPERIENCE of a moroccan addiction departement

**DOI:** 10.1192/j.eurpsy.2023.1362

**Published:** 2023-07-19

**Authors:** A. Tounsi, F. Azraf, N. El Moussaoui, M. Sabir, F. Elomari

**Affiliations:** 1 Psychiatric; 2 Addiction, Arrazi university psychiatric hospital, Sale, Morocco

## Abstract

**Introduction:**

Inhalants are volatile psychoactive compounds whose effect varies from disorientation, excitement, euphoria to hallucinations. Different opinions have been raised concerning the relationship between inhalant use and psychosis and several publications have studied the incidence of psychotic disorders in the context of inhalant use. These studies concluded that using inhalants was independently associated with the development of psychosis

**Objectives:**

our aim is to determine the demographic and psychiatric profile of inhalant users previously hospitalized in our department

**Methods:**

This is a retrospective descriptive study carried out by analyzing hospitalization records in the addictology department of the psychiatric university hospital Ar-Razi in Salé over a period of one year (from August 2020 to August 2021). The diagnoses are established according to the DSM 5 diagnostic criteria.

**Results:**

Seventeen patients, inhalant users, were recruited after chart review, including 5 women and 11 men (68.7 %). The average age was 24.7 years (16; 41). The majority of the patients were single (81.2%), 62.5 % had a secondary education and 62.5 % were unemployed.

The psychiatric evaluation showed that 87% of these patients had a history of incarceration, 50% had a diagnosis of schizophrenia, 12.5% had bipolar disorder and 14.2% had a cluster B personality disorder (DSM 5).

The average age of onset of the addictive disorder in this population was 14.4 years and the entire sample was polyaddictive.

**Conclusions:**

More than half of our sample had psychosis associated with their inhalant use disorder. these results are consistent with literature data.

**Disclosure of Interest:**

None Declared

